# 

**Published:** 1844-09

**Authors:** 


### WESTERN JOURNAL

OF

### MEDICINE AND SURGERY.

### EDITORS.

---

DANIEL DRAKE, M. D.,

PROFESSOR OF PATHOLOGY AND THE PRACTICE OF MEDICINE IN THE MEDICAL INSTITUTE OF LOUISVILLE:

LUNSFORD P. YANDELL, M. D.,

PROFESSOR OF CHEMISTRY AND PHARMACY IN THE SAME:

THOMAS W. COLESCOTT, M. D.

BOOKS RECEIVED.

Cyclopædia of Practical Medicine, parts viii., ix., x. Dunglison's Elements of Hygiene Wilson's Human Anatomy. The Superstitions connected with Medicine and Surgery, by Thomas Joseph Pettigrew, F.R.S., &c. Moreau's Practical Treatise on Midwifery. Druitt's Modern Surgery. The American Journal of Insanity, No. I. We have barely room to acknowledge the receipt of these works, at present. They will be noticed hereafter.

